# To Be Cytosolic or Vacuolar: The Double Life of *Listeria monocytogenes*

**DOI:** 10.3389/fcimb.2018.00136

**Published:** 2018-05-15

**Authors:** Hélène Bierne, Eliane Milohanic, Mounia Kortebi

**Affiliations:** Epigenetics and Cellular Microbiology Team, Micalis Institute, Institut National de la Recherche Agronomique, AgroParisTech, Université Paris-Saclay, Jouy-en-Josas, France

**Keywords:** infectious diseases, pathogenesis, persistence, persisters, VBNC, antibiotic resistance

## Abstract

Intracellular bacterial pathogens are generally classified into two types: those that exploit host membrane trafficking to construct specific niches in vacuoles (i.e., “vacuolar pathogens”), and those that escape from vacuoles into the cytosol, where they proliferate and often spread to neighboring cells (i.e., “cytosolic pathogens”). However, the boundary between these distinct intracellular phenotypes is tenuous and may depend on the timing of infection and on the host cell type. Here, we discuss recent progress highlighting this phenotypic duality in *Listeria monocytogenes*, which has long been a model for cytosolic pathogens, but now emerges as a bacterium also capable of residing in vacuoles, in a slow/non-growing state. The ability of *L. monocytogenes* to enter a persistence stage in vacuoles might play a role during the asymptomatic incubation period of listeriosis and/or the carriage of this pathogen in asymptomatic hosts. Moreover, persistent vacuolar *Listeria* could be less susceptible to antibiotics and more difficult to detect by routine techniques of clinical biology. These hypotheses deserve to be explored in order to better manage the risks related to this food-borne pathogen.

## Background: the prototype of a cytosolic and disseminating bacterium

**Figure 1:**
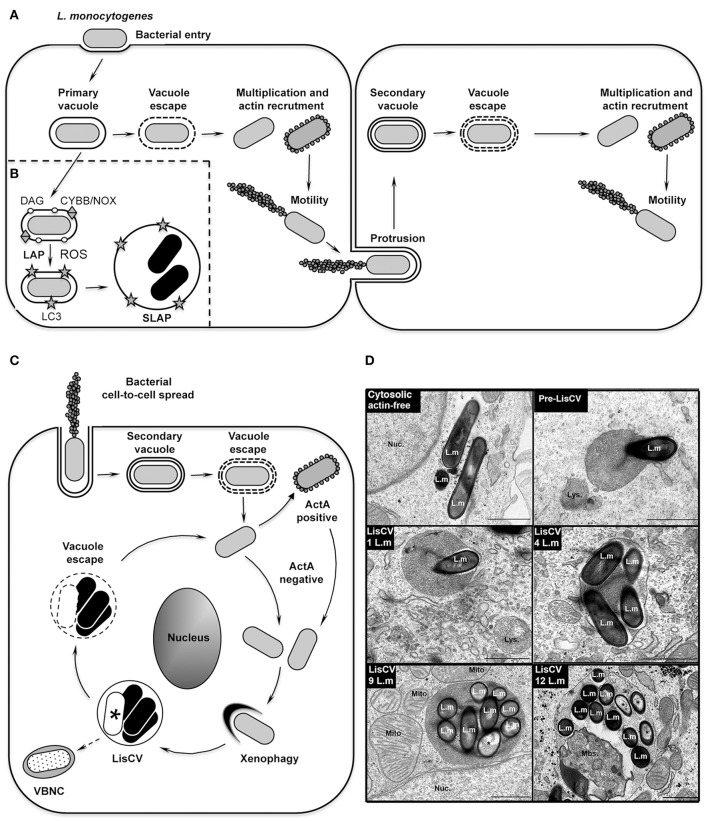
The intracellular life cycle of *L. monocytogenes*. **(A)** Intracellular invasion and dissemination of *L. monocytogenes* in mammalian cells (adapted from Portnoy, 2012). After bacterial entry into the host cell and transient residence within a primary vacuole, bacteria escape into the cytoplasm, multiply and induce expression of the actin-polymerizing factor ActA. Actin polymerization promotes bacterial motility and cell-to-cell spread via the generation of membrane protrusions from the primary infected cell to neighboring cells. After the resolution of these protrusions into double-membrane secondary vacuoles, from which the bacteria escape, a new cycle of infection is initiated. **(B)** Model of SLAP formation in murine macrophages (adapted from Lam et al., 2013). After bacterial phagocytosis, subpopulations of bacteria expressing low amounts of the cytolysin LLO remain in phagosomes. Diacylglycerol accumulates on these phagosomes, as a result of bacterial (PLC) and host (PLD and PPAP2A) enzymatic activities. DAG accumulation stimulates activation of the CYBB/NOX2 NADPH oxidase and the production of reactive oxygen species (ROS), which subsequently induce recruitment of LC3 to the phagosome. This LC3-associated-phagocytosis (LAP) process is not harmful for *Listeria* and promotes formation of SLAPs, in which bacteria enter a slow/non-replicative state (black bacteria). **(C)** Model of LisCV formation in human hepatocytes and trophoblast cells (adapted from Kortebi et al., 2017). Following the active stage of bacterial cell-to-cell spread (as in **A**), bacteria do not re-express ActA after escape from the secondary vacuole, or stop expressing ActA after a transient ActA-positive cytosolic stage. ActA-free bacteria multiply in the cytosol and are captured by a xenophagy-like process, forming *Listeria*-containing vacuoles (LisCV). In these lysosome-like compartments, subpopulations of bacteria resist stress and degradation and enter a slow/non-replicative state (black bacteria), while others are sensitive to stress and die (white bacteria with a “^*^”). Upon unidentified stimuli, bacteria may egress from vacuoles and re-initiate a novel cycle of actin-polymerization following re-expression of ActA. In absence of a reactivation signal, wild type bacteria may behave like Δ*actA* bacteria, which enter the VBNC state (punctuated bacteria). **(D)** TEM micrographs illustrating different steps of LisCV formation in JEG3 cells infected for 72 h. Shown are the actin-free cytosolic stage, the capture of a bacterium by uncharacterized membranous electron-dense compartment (“pre-LisCV”) and the vacuolar stage with LisCVs containing different numbers of bacteria. Damaged bacteria are indicated with a “^*^”. L.m, *L. monocytogenes*; Nuc., nucleus; Lys., secondary lysosomes; Mito., mitochondria; Mbs., membranous intravacuolar structures (Adapted from Kortebi et al., 2017).

*Listeria monocytogenes* is the etiologic agent of listeriosis, a severe foodborne disease leading to blood, brain or fetal infections in humans and many animal species (Allerberger and Wagner, 2010; Dhama et al., 2015). The link between the virulence of this pathogen and its faculty to invade mammalian cells was discovered in the 1960–1970s, with the pioneering work of Mackaness showing that *Listeria* can replicate in macrophages in mice (Mackaness, 1962), and of Racz and colleagues, documenting the *Listeria* infection process in epithelial tissues of guinea pigs (Rácz et al., 1970, 1972, 1973). In the 1980–1990s, the ability to model bacterial infections in tissue-cultured cells led to the characterization of the *L. monocytogenes* intracellular infectious process (Portnoy et al., 1992; Portnoy, 2012). It was established that following phagocytosis or receptor-mediated endocytosis, bacteria disrupt the invasion vacuole and enter the cytosol, where they replicate and use actin polymerization to spread from cell-to-cell (Figure 1A). Another landmark discovery of the 1990s was the identification of the bacterial determinants of *L. monocytogenes* entry and movement in the cytosol (Portnoy et al., 1992). In particular, *Listeria* escapes primary and secondary vacuoles using the pore-forming toxin Listeriolysin O (LLO) in cooperation with two phospholipase C (PI-PLC and PC-PLC), while the actin assembly-inducing protein ActA drives bacterial propulsion by inducing actin polymerization on the bacterial surface. These groundbreaking findings laid the foundation of the field of “Cellular Listeriology,” making *L. monocytogenes* one of the best-characterized intracytosolic pathogens. The bacterial effectors used by *Listeria* to achieve a successful infection (about 40 have been identified, so far), as well as the host cell processes manipulated by this pathogen, have been widely discussed in recent reviews (Lebreton et al., 2016; Radoshevich and Cossart, 2018). The metabolic adaptations to cytosolic life, allowing this pathogen to replicate and resist host-derived stresses and defenses, is also well documented (Chen et al., 2017).

However, most of these discoveries were generated by studying the early stages of the cellular infection process; the long-term fate of cytosolic *Listeria* received little attention. By studying longer-term infections in human epithelial cells, we recently observed an astonishing change in the *L. monocytogenes* intracellular lifestyle in hepatocytes and trophoblast cells. After 2–3 days of infection, bacteria are engulfed in vacuoles, termed *Listeria*-containing vacuoles (LisCVs), where they enter a slow/non-growing state (Kortebi et al., 2017). Other studies had previously revealed that in murine macrophages *Listeria* can also enter a quiescent state within vacuoles, called Spacious *Listeria*-containing vacuoles (SLAPs) (Birmingham et al., 2008; Lam et al., 2013), but by a mechanism coupled to phagocytosis at the onset of infection. Here, we aim to compare these two types of vacuolar niches and to integrate them into the intracellular infectious process of *L. monocytogenes*. We will also discuss their physiological relevance in tissues and potential impact on human or animal health.

## The SLAP, a niche for *L. monocytogenes* persistence in phagocytic cells

The idea that *L. monocytogenes* could occupy distinct intracellular habitats emerged with the study of chronic infections in severe combined immunodeficiency (SCID) mice, which lack adaptive immunity. After 3 weeks of infection, *Listeria* bacteria were present in granulomatous lesions of the liver, but surprisingly not as cytosolic forms. Transmission electron microscopy (TEM) studies of these lesions identified groups of bacteria within large single-membrane vacuoles in macrophages (Bhardwaj et al., 1998). This puzzling result opened the possibility that this microorganism, despite being so well adapted to cytosolic life, could have another intracellular fate. Brumell and colleagues further showed that these Spacious *Listeria*-containing vacuoles (SLAPs) were positive for lysosomal-associated membrane protein 1 (LAMP1), indicating that they fuse with endocytic compartments (Birmingham et al., 2008). The mechanism of SLAP formation was then detailed in murine macrophages cultured *in vitro*. SLAPs are phagosomes that do not mature into phagolysosomes, entrapping a subset of bacteria that produce low amounts of the cytolysin LLO (Birmingham et al., 2008). Some evidence suggests that the LC3-associated phagocytosis pathway (LAP) is involved in the formation of these compartments (Figure 1B). LAP is a non-canonical autophagy pathway involved in the maturation of single membrane phagosomes (Schille et al., 2018). This mechanism is associated with the production of reactive oxygen species (ROS) by a NADPH oxidase, followed by the lipidation of the autophagy protein LC3 on the phagosomal membrane. During phagocytosis of *L. monocytogenes*, LC3 is indeed recruited to phagosomes by a mechanism requiring the activity of the CYBB/NOX2/NADPH oxidase and subsequent ROS production (Lam et al., 2013). Activation of the NADPH oxidase involves the accumulation of diacylglycerol on the phagosome, as a result of both bacterial (PLC) and host (PLD and PPAP2A) phospholipase activities. It is proposed that subpopulations of *L. monocytogenes* hijack the LAP pathway to their advantage, avoiding the ultimate degradative step of this pathway, hence generating a vacuolar niche for persistent infection in phagocytes (Lam et al., 2013). However, the mechanisms that prevent a SLAP from becoming a destructive phagolysosome remain to be elucidated.

## The LisCV, a niche for *L. monocytogenes* persistence in a subset of epithelial cells

Recently, we described a new type of *Listeria*-containing vacuole (LisCV) in human hepatocytes and trophoblast cells. In these cells, *L. monocytogenes* progressively ceases to polymerize actin and, after 2–3 days of infection, cytosolic bacteria are captured in membrane structures, forming LisCVs (Figure 1C; Kortebi et al., 2017). Like SLAPs, LisCVs are single-membrane LAMP1-positive vacuoles, in which bacteria enter a slow/non-replicative state. However, unlike SLAPs, LisCVs are acidic and positive for the lysosomal marker cathepsin D, indicating that these compartments fuse with lysosomes. LisCVs are actually partially degradative for *L. monocytogenes*, but a majority of bacteria remain intact (Kortebi et al., 2017). Indeed, *L. monocytogenes* has powerful mechanisms to withstand various stresses, including low pH conditions (Ryan et al., 2008), which could favor its adaptation to the most inhospitable intracellular conditions, such as those found in lysosomes, as described for the vacuolar pathogen *Coxiella burnetti* (Kohler and Roy, 2015).

The formation of LisCVs leaves several questions open, particularly its link with xenophagy, a selective autophagy process that restricts the growth of intracellular microbes (Bauckman et al., 2015). Immunofluorescence microcopy and TEM studies (Figure 1D) provide evidence that LisCVs are formed by re-entry of *L. monocytogenes* into a membrane-associated compartment after a cytosolic step (Kortebi et al., 2017). This is reminiscent of a phenomenon described for the bacterium *Francisella tularensis*, which escapes from phagosomes and is engulfed in vacuoles following a transient cytosolic phase in certain cells (Checroun et al., 2006). However, while *Francisella*-containing vacuoles (FCVs) are delimited by a double-membrane and are targeted by the autophagy protein LC3, LisCV are single membrane LC3-negative vacuoles, and the knockdown of canonical autophagy factors does not prevent their formation (Kortebi et al., 2017). LisCVs could thus be formed through a yet-unknown xenophagy process that allows eukaryotic cells to sequester microbial invaders from the cytosol. Noteworthy, *Mycobacterium marinum* (an actin-polymerizing bacterium, like *L. monocytogenes)* can also be captured in lysosome-like vacuoles by a pathway independent of canonical autophagy (Collins et al., 2009).

On the bacterial side, the formation of LisCVs is concomitant to the loss of the actin-nucleating protein ActA and the arrest of actin polymerization at the bacterial surface. The bacteria thus exposed could become accessible to non-canonical autophagy factors, in the same way that ActA deficiency allows the targeting of *Listeria* by canonical autophagy proteins early on in infection (Yoshikawa et al., 2009; Mitchell et al., 2015). The mechanism behind the disappearance of ActA during prolonged infections is not yet elucidated. It should be noted that ActA is proteolytically cleaved by the bacterial metalloprotease Mpl during acidification of secondary vacuoles and there is a delay before ActA is re-synthesized after bacterial escape from these vacuoles (Robbins et al., 1999; Alvarez and Agaisse, 2016). LisCVs formed in hepatocytes and trophoblast cells could thus result from entrapment of cytosolic *Listeria* with cleaved, non-functional ActA. Alternatively, the *actA* gene could be shut down after long-term infection. Indeed, it is tempting to speculate that, for prolonged vacuolar maintenance, the virulence gene cluster that carry *actA*, as well as the genes encoding the bacterial lytic enzymes (LLO, PI-PLC, and PC-PLC) and the transcriptional activator PrfA (Vázquez-Boland et al., 2001), is down-regulated. Additional research is needed to provide evidence for such down-regulation. Among other regulators of the phenotypic switch between bacterial dissemination and persistence, one can think of second bacterial messengers, such as cyclic dimeric GMP (c-di-GMP). This molecule is known for its key role in bacterial lifestyle transitions (Valentini and Filloux, 2016). The components of the c-di-GMP signaling pathway have been identified in *L. monocytogenes* (Chen et al., 2014), opening an interesting research perspective on its relationship with *Listeria* persistence.

LisCVs remain intact in dividing cells at different stages of mitosis (Kortebi et al., 2017), in the same way that lysosomes do not disintegrate during mitosis, but are partitioned as separate vesicles (Bergeland et al., 2001). This ability to segregate in daughter cells could be a way for persistent *Listeria* to spread during epithelial cell renewal, which promotes tissue repair after acute infection. By studying the fate of LisCVs during subculture of host cells *in vitro*, we observed these vacuoles in cell progenies. However, in certain cell populations *Listeria* escaped from LisCVs and returned to an active phase of proliferation and dissemination (Kortebi et al., 2017). From this observation, we propose that *L. monocytogenes* has evolved mechanisms to regulate the phenotypic switch between cytosolic and vacuolar stages in epithelial cells.

## The intracellular viable but non-culturable state of *L. monocytogenes*

Several bacterial species with vacuolar lifestyles can persist for long periods in their mammalian hosts, if they are not eradicated during the resolution of the symptomatic infection or after antibiotic treatments (Monack et al., 2004). A particular form of long–term persistence occurs when bacteria reach the viable but non-culturable (VBNC) state characterized by a loss of culturability on routine agar, which impairs the detection of VBNC forms by conventional plate count techniques (Li et al., 2014). Many pathogens, including *L. monocytogenes* (Besnard et al., 2002; Cappelier et al., 2005; Dreux et al., 2007; Lindbäck et al., 2010), have been shown to enter the VBNC state *in vitro*, in response to environmental stresses (Ramamurthy et al., 2014). Yet, this state has rarely been demonstrated in host cells. The best-known paradigm is the VBNC status of *Mycobacterium tuberculosis* in human lung cells, which promotes the asymptomatic carriage of this pathogen (or “latency”) in more than two million people worldwide (Gengenbacher and Kaufmann, 2012). We recently uncovered that *L. monocytogenes* can also enter the VBNC state within epithelial cells, when the *actA* gene is kept inactive. Moreover, these VBNC forms can be passively propagated during mitosis (Kortebi et al., 2017). These results open the possibility that *L. monocytogenes* could generate intracellular dormant forms that may not be detected by diagnostic tests based on bacterial growth from body tissue or fluid samples.

## Intracellular phenotypes of *L. monocytogenes* in mammalian tissues

**Figure 2:**
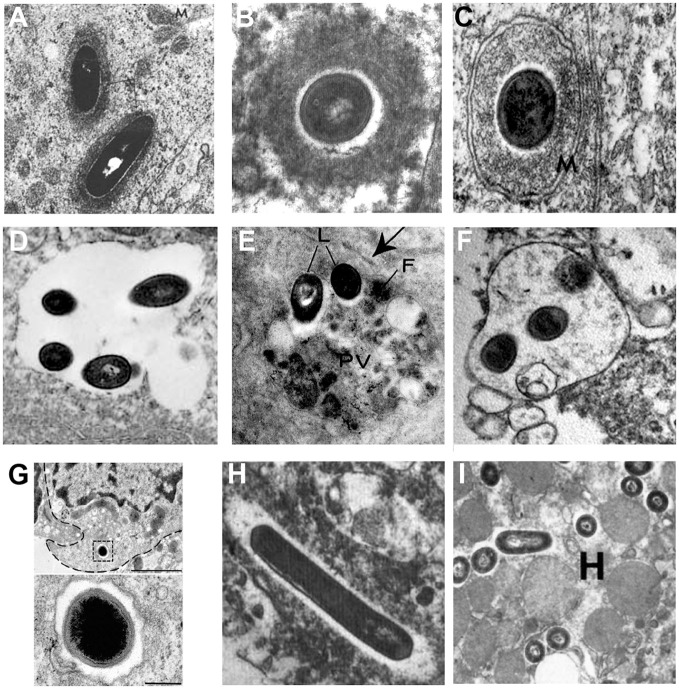
Different stages of the intracellular life of *L. monocytogenes* observed by TEM in mammalian infected tissues. Images are TEM micrographs reproduced from *in vivo* studies, with permission. **(A)** Two *Listeria* surrounded by a fibrillar cloud of actin filaments in an epithelial cell from the jejunum of a guinea pig infected for 22 h [© Rácz et al. (1972). Originally published in Laboratory Investigation]. **(B)** A bacterium surrounded by a cloud of actin filaments in a brain stem cell isolated from a patient who has died from neurolisteriosis [© Kirk (1993). Originally published in Ultrastructural Pathology]. **(C)** A *Listeria* within a two-membrane bound vacuole in an epithelial cell from the urinary bladder of a guinea pig infected for 24 h [© Rácz et al. (1973). Originally published in Virchows Archiv, B Cell Pathology]. **(D)** A spacious single membrane-bound vacuole enclosing four *Listeria* in a macrophage from the liver of a SCID mouse infected for 21 days [© Bhardwaj et al. (1998). Originally published in The Journal of Immunology]. **(E)** Two *Listeria* (L) within a phagolysosome-like vacuole (PV) containing heterogeneous material and ferritin **(F)** in a macrophage from the spleen of a mouse infected for 72 h [© Armstrong and Sword (1966). Originally published in Journal of Bacteriology]. **(F)** A vacuole enclosing three *Listeria* and heterogeneous material in an intra-axonal neutrophil from the brain stem of a ruminant with neurolisteriosis [© Henke et al. (2015). Originally published in Infection and Immunity]. **(G)** A bacterium enclosed in a transcytosis vacuole close to the basal membrane, in a murine intestinal goblet cell. The bacterium is shown at a higher magnification below. Experiments were performed in a transgenic mouse expressing humanized E-cadherin, a key receptor for *Listeria* entry into epithelial cells. Exocytosis has been blocked to prevent the exocytosis of the vacuole in the lamina propria [© Nikitas et al. (2011). Originally published in Journal of Experimental Medicine]. **(H)** Two bacteria free in the cytosol within an electron-transparent halo in a brain stem macrophage isolated from a patient who has died from neurolisteriosis [© Kirk (1993). Originally published in Ultrastructural Pathology]. **(I)** Several *Listeria* free in the cytosol in a hepatocyte **(H)** from the liver of a mouse infected for 24 h [© Gaillard et al. (1996). Originally published in Journal of Experimental Medicine].

Intracellular lifestyles of *L. monocytogenes* have been widely dissected in tissue-cultured cells, but few studies have confirmed the subcellular localization of *Listeria* during *in vivo* infection in mammalian species. Illustrative examples based on TEM studies are presented in Figure 2. Observations of *Listeria* surrounded by “clouds” of dense fibrillar material document the actin-based motility stage. These structures have been observed in epithelial cells of infected guinea pigs (Rácz et al., 1972; Figure 2A) and in brain cells from neurolisteriosis cases in humans (Kirk, 1993; Figure 2B) and in ruminants (Henke et al., 2015). The dissemination stage is highlighted by membrane protrusions and double-membrane secondary vacuoles detected in epithelial cells of infected guinea pigs (Rácz et al., 1972, 1973; Figure 2C). As mentioned above, the persistence stage in vacuoles was discovered with the observation of SLAPs in hepatic macrophages in SCID mice (Bhardwaj et al., 1998; Birmingham et al., 2008; Figure 2D). In contrast, evidence for the formation of LisCVs *in vivo* are still lacking. Yet, it has to be noted that LisCVs appear in primary human hepatocytes cultured *in vitro* (Kortebi et al., 2017). Although the liver plays a key role in the clearance of *L. monocytogenes* infections, liver disorders are rare during listeriosis and the hepatic phase of listeriosis is mainly asymptomatic (Scholing et al., 2007). Whether the liver is a reservoir for vacuolar *Listeria* in hepatocytes, as shown in macrophages, clearly deserves more investigation. Regarding other tissues, uncharacterized types of single membrane-bound vacuoles sequestering *Listeria* have been described in splenic macrophages in mice (Armstrong and Sword, 1966; Figure 2E) and intra-axonal neutrophils of ruminants with rhombencephalitis (Henke et al., 2015; Figure 2F). These vacuoles contain intact bacteria and heterogeneous cellular material of unknown origin. The fate of these vacuoles in the long term is totally unknown. In the gut, an original type of vacuole has been described in intestinal goblet cells. Here, *Listeria* transcytoses in the entry vacuole not to persist but to rapidly cross the intestinal barrier (Figure 2G; Nikitas et al., 2011). Of note, *Listeria* uses alternate mechanisms to cross this barrier (Pentecost et al., 2006; Melton-Witt et al., 2012; Drolia et al., 2018).

The stage of *Listeria* free within the cytosol (i.e., associated neither to actin nor membranes) has been documented for instance in a brain macrophage from a human neurolisteriosis case (Kirk, 1993; Figure 2H), as well as in hepatocytes of mice infected for 24 h (Gaillard et al., 1996; Figure 2I). These bacteria could have just escaped primary or secondary vacuoles, before starting the actin polymerization step. Some might be targeted for sequestration in persistence vacuoles, a possible fate for actin-free bacteria that cannot be excluded.

These hypotheses open many avenues for additional research on vacuolar niches for *L. monocytogenes* in different target organs.

## Intracellular persistence and asymptomatic carriage of *listeria*: a relationship?

Listeriosis is a food-borne disease. Outbreaks triggered by highly contaminated food products affect large groups of people but represent only a small percentage of all listeriosis cases (Ebel et al., 2016). Listeriosis is essentially sporadic and the source of contaminated food is difficult to identify. Data suggests that oral exposure to low doses of *L. monocytogenes* is relatively common (Hof, 2001; Grif et al., 2003). Nevertheless, the incidence of listeriosis remains low (1–10 cases per million per year) (Goulet et al., 2012). This may seem paradoxical, given the sophisticated mechanisms used by *L. monocytogenes* to invade, proliferate and survive in the host (Lebreton et al., 2016; Radoshevich and Cossart, 2018). The rarity of invasive listeriosis appears to be mainly due to cell-mediated immunity, which is highly protective against this pathogen. However, with regards to the persistence phase discussed here, one should now question the contribution of non-replicative vacuolar forms of *Listeria* in a benign carriage of this pathogen.

People who develop invasive listeriosis generally have a weakened immune system related to aging, underlying diseases and/or immunosuppressive therapies (Friesema et al., 2015; Charlier et al., 2017). Pregnancy-related suppressed cell-mediated immunity and placental tropism of *L. monocytogenes* also make this illness dangerous for fetuses, while mothers are mostly asymptomatic. The incubation period of listeriosis can be long, especially in cases associated with pregnancy, for which it can last up to 3 months (Goulet et al., 2013). Compared with other foodborne pathogens, this asymptomatic incubation is unusually extended. Could a vacuolar stage play a role in this incubation phase? It is a question worth asking. *L. monocytogenes* persistent forms might also be implicated in a few recurrent listeriosis cases (McLauchlin et al., 1991; Sauders et al., 2001; Kleemann et al., 2009), as well as in recurrent early pregnancy loss, a hypothesis raised a long time ago (Rappaport et al., 1960; Gray and Killinger, 1966; Romaña et al., 1989), but which is still a matter of debate (Lamont et al., 2011). Finally, the existence of silent *L. monocytogenes* infections could be highly relevant in livestock production, with bacterial reservoirs remaining undetected because of asymptomatic carriage.

Antibiotherapy of invasive listeriosis is still an issue, as many patients succumb to this infection despite antibiotic administration (Charlier et al., 2017). Are intravacuolar *Listeria* a possible cause for this antibiotic therapy failure? The primary treatment for listeriosis consists of a combination regimen of ampicillin and gentamicin (Allerberger and Wagner, 2010). Ampicillin, which targets the cell wall synthesis machinery, could be poorly effective against non-growing *Listeria*. Moreover, gentamicin does not seem to be effective against *Listeria* within LisCVs (Kortebi et al., 2017), consistent with the observation that aminoglycosides are almost devoid of activity at the acidic pH of lysosomes (Maurin and Raoult, 2001). Consequently, there is a need to carefully examine whether vacuolar *Listeria* generate persisters tolerant to antibiotics, as described for other pathogen species (Fisher et al., 2017; Van den Bergh et al., 2017).

## Concluding remarks

Vacuoles are underexplored niches in *L. monocytogenes* pathogenesis research and there are fascinating biological questions to be resolved concerning these little-studied vacuolar stages. To enable further progress in the *in vivo* characterization of *L. monocytogenes* intravacuolar niches of persistence, several obstacles must be overcome. First, persistent bacteria are likely to be present in very low numbers in tissues, and if in the dormant VBNC state, not detectable by growth in conventional culture media. Additionally, if vacuolar persistence somehow changes antigen-presentation and/or secretion of immune mediators, cells containing these vacuolar forms may poorly attract immune cells, which are commonly used as a landmark for infection foci. Other technical difficulties will be encountered down the road, particularly in the choice of the host to be studied. Obtaining tissue samples from human asymptomatic carriers will be challenging. The murine model of listeriosis may not be optimal for investigating the persistence of *L. monocytogenes* in epithelial tissues due to the low internalization rate of *Listeria* in murine epithelial cells (Lecuit, 2007) and to differences in mucosal immunity between mice and humans (Gibbons and Spencer, 2011). Therefore, there is a need to develop sensitive detection methods and test different animal models in order to locate both persistent *Listeria* and their host cells. There is significant potential to improve disease control and food safety if this vacuolar phase of the disease cycle can be detected, studied and disrupted.

## Author contributions

HB wrote the manuscript. EM and MK contributed to the final version of the manuscript.

### Conflict of interest statement

The authors declare that the research was conducted in the absence of any commercial or financial relationships that could be construed as a potential conflict of interest.
